# Effect of red blood cell transfusion on inflammation, endothelial cell activation and coagulation in the critically ill

**DOI:** 10.1111/vox.13125

**Published:** 2021-07-01

**Authors:** Lisa van Manen, Maike E. van Hezel, Margit Boshuizen, Marleen Straat, Angelique M. E. de Man, Charlotte Dekimpe, Karen Vanhoorelbeke, Robin van Bruggen, Nicole P. Juffermans

**Affiliations:** ^1^ Department of Intensive Care Medicine and Laboratory of Experimental Intensive Care and Anesthesiology Amsterdam UMC, Location VUmc, University of Amsterdam Amsterdam The Netherlands; ^2^ Department of Blood Cell Research, Sanquin research and Landsteiner Laboratory University of Amsterdam Amsterdam The Netherlands; ^3^ Department of Intensive Care Medicine Amsterdam UMC, Location VUmc Amsterdam The Netherlands; ^4^ Laboratory for Thrombosis Research KU Leuven Campus Kulak Kortrijk Kortrijk Belgium; ^5^ Department of Intensive Care Medicine OLVG Hospital Amsterdam The Netherlands

**Keywords:** coagulation, critically ill patients, endothelial activation, inflammation, RBC transfusion

## Abstract

**Background and Objectives:**

Red blood cell (RBC) transfusion is a frequently applied intervention in an intensive care unit. However, transfusion is associated with adverse outcomes including organ failure and thrombo‐embolic events. Mechanisms of these effects are not known but may be related to activation of the endothelium or of the coagulation or inflammatory system. We hypothesized that a RBC transfusion in the critically ill would result in further activation of these systems.

**Materials and Methods:**

In 74 non‐bleeding critically ill patients receiving one RBC unit, markers of inflammation, endothelial cell activation and coagulation were measured before transfusion, at 1 h after transfusion and 24 h after transfusion. The impact of disease severity of the recipient on these changes was assessed by comparing septic and non‐septic patients (according to sepsis‐3 definition) and by correlation of biomarkers with the sequential organ failure assessment (SOFA) score.

**Results:**

Levels of von Willebrand Factor (vWF), soluble ICAM‐1, soluble thrombomodulin, fibrinogen and d‐dimer were already high at baseline, whereas ADAMTS13 levels were low. VWF levels increased significantly 24 h after RBC transfusion (median 478% (338–597) vs. 526% (395–623), *p* = 0.009). The other biomarkers did not change significantly. Post transfusion change was not dependent on the presence of sepsis and was not correlated with SOFA score.

**Conclusion:**

RBC transfusion in critically ill patients was associated with an increase in circulating vWF levels, suggesting a further increase in activation of the endothelium, a finding that was independent of the presence of sepsis or organ injury level.

## INTRODUCTION

More than 1 out of four patients in an intensive care unit (ICU) receive a red blood cell (RBC) transfusion during their admission, rendering RBC transfusion one of the most frequently applied interventions at the ICU [1]. However, RBC transfusion is associated with adverse outcomes including organ failure and thrombo‐embolic events, in particular in the critically ill [2, 3, 4, 5]. The mechanisms responsible for these adverse events are not fully understood.

Critically ill patients are often in an inflammatory state in which the endothelium and coagulation system are already activated before transfusion [6]. The presence of an inflammatory state in the recipient has been shown to be a risk factor for the development of transfusion related acute lung injury (TRALI) [5]. Also, in patients with an inflammatory state that do not develop full‐blown TRALI, an extra ‘hit’ by the RBC transfusion may exacerbate inflammation, potentially resulting in transfusion related adverse events [7, 8, 9, 10]. Since the vascular endothelium and glycocalyx are among the first that interact with the donor RBCs after transfusion, these structures might also play a role in the pathophysiology. RBC transfusion is associated with increased biomarker levels of endothelial cell activation in haematological and paediatric patients [11, 12]. Endothelial cell activation can lead to increased endothelial permeability with neutrophil extravasation and capillary leakage, resulting in organ injury [13]. Activation of the endothelium also activates the coagulation system, resulting in (micro)thrombus formation [14, 15]. Therefore, the vascular endothelium and activation of the coagulation system might be involved in the adverse events of RBC transfusion.

The aim of this study is to investigate the effect of RBC transfusion on several biomarkers of inflammation, endothelial cell activation and coagulation in adult critically ill patients (Table 1). We hypothesized that a RBC transfusion in the critically ill results in further activation of the vascular endothelium and also in activation of the coagulation system. Since sepsis is a risk factor for developing transfusion‐ associated adverse events, we expected a greater effect in septic patient compared to non‐septic patients [5].

**TABLE 1:** Measured biomarkers and their function

Biomarker	Function
*Markers of inflammation*	
IL‐6	Pro‐inflammatory cytokine
TNF‐alpha	Pro‐inflammatory cytokine
*Markers of endothelial cell activation*	
Soluble syndecan‐1	Marker of glycocalyx degradation
Soluble thrombomodulin	Marker of endothelial cell damage
Soluble ICAM‐1	Marker of endothelial cell activation
Von Willebrand Factor	Marker of endothelial ceactivation and involved in coagulation cascade
ADAMTS13	Responsible for cleavage of ultra large multimers of vWF
*Markers of coagulation*	
D‐dimer	Fibrin degradation product
Fibrinogen	Precursor of fibrin, converted into fibrin during clot formation

Abbreviations: ADAMTS‐13, a disintegrin and metalloproteinase with a thrombospondin type 1 motif, member 13; ICAM‐1, intercellular adhesion molecule 1; IL‐6, interleukine‐6; TNF, tumour necrosis factor.

## MATERIALS AND METHODS

### Study design

A prospective, observational study was conducted on the intensive care units of two tertiary hospitals in the Netherlands between 2011–2015 and 2017–2018. Due to logistical problems the study was temporarily interrupted between 2015 and 2017. The study was ethically approved by the medical ethical committee of the Amsterdam University Medical Centre (NTR 6596; NL61833.018.17). Written informed consent was obtained from all participants or their legal representatives. Non‐bleeding patients receiving one RBC unit to correct for anaemia were included. Patients without an indwelling arterial catheter were excluded. Patients were lost to follow‐up when indwelling arterial catheter was removed. Local transfusion protocol dictated a transfusion trigger of 7.0 g/dl in the general ICU population and 8.0 g/dl for those with acute coronary syndrome. The RBC unit was plasma reduced, leucocyte depleted and stored in additive solution (SAGM). The average volume of RBC unit is 270–290 ml. The RBC unit was produced according to the national standards of Sanquin Blood Supply Foundation, Amsterdam, the Netherlands. Sepsis was defined as a sequential organ failure assessment (SOFA) score of at least 2 and a suspected or proven infection treated with antibiotics according to the Sepsis‐3 criteria [16].

### Study procedures

Blood was drawn from an indwelling arterial catheter into EDTA and citrate tubes before transfusion, within 1 h after the completion of the transfusion and 24 and 48 h after transfusion. Since we expected the effect of the RBC transfusion within 24 h after RBC transfusion we measured biomarkers directly after and 24 h after transfusion. We did not expect an effect of transfusion after 48 h, therefore we used this sample only to measure the vWF antigen. A further increase after 48 h could indicate a natural upward course of vWF during ICU admission.

A complete blood count was done using EDTA blood (Sysmex XN9000, Etten‐Leur, The Netherlands). Thereafter blood was centrifuged for 20 min at 1500G. Prothrombin time (PT), activated partial thromboplastin time (APTT), fibrinogen and d‐dimer levels were determined in citrate plasma (CS2500, Siemens Healthcare GmbH, Germany). Plasma samples were frozen at −80°C until further analysis.

Measured biomarkers are listed in Table 1. Soluble Syndecan‐1, soluble ICAM‐1, soluble thrombomodulin (TM), IL‐6 and TNF‐alpha were measured in EDTA plasma with a custom designed human premixed multi‐analyte luminex assay (Luminex R&D Systems Inc., Minneapolis, MN, USA) as described by the manufacturer. Von Willebrand Factor (vWF) antigen was measured in citrate plasma with ELISA (DAKO, Glostrup, Denmark).

Human ADAMTS13 (a disintegrin and metalloproteinase with a thrombospondin type 1 motif, member 13) antigen levels were determined using a monoclonal antibody‐based human ADAMTS13 antigen ELISA, as previously described [17, 18, 19]. Microtiter plates were coated with the monoclonal mouse anti‐human ADAMTS13 antibody 3H9 [17, 18, 20] overnight at 4°C (5 μg/ml in carbonate/bicarbonate buffer) and subsequently blocked with phosphate‐buffered saline (PBS) with 3% dried milk powder for 2 h at room temperature (RT). Next, plasma samples (starting dilution of 1/50) were added in a 1.5 over 2.5 dilution series and incubated for 1.5 h at 37°C. Captured ADAMTS13 was detected using a mixture of biotinylated mouse anti‐human ADAMTS13 antibodies 17G2 and 19H4 [17, 18, 21] (1.5 μg/ml each, incubation for 1 h at RT) followed by horseradish peroxidase (HRP)‐labelled streptavidin (1/10,000; Roche Diagnostics, Mannheim, Germany) (incubation for 1 h at RT). The colorimetric reaction was initiated by addition of o‐phenylenediamine (OPD) and H_2_O_2_, stopped with 4 M sulfuric acid, and the absorbance was measured at 490 nm. A dilution series of a normal human plasma pool (NHP, plasma from ≥20 healthy donors, set at 100%) was used as a reference, from which the ADAMTS13 antigen levels were interpolated.

### Statistical analysis

Variables are presented as means with standard deviation or as medians with interquartile ranges when not normally distributed, and categorical data are presented as number with percentage. For continuous data, comparisons between two groups were made using the *t*‐test or when not normally distributed the Mann–Whitney U test. More than two groups were compared using the Kruskal Wallis test. For categorical data, the Chi‐squared test was used. Paired data analysis were done with the Friedman test and Wilcoxon signed rank test. Correlation coefficient between the delta of the biomarkers and SOFA score was calculated using the Spearman correlation. A P‐value of less than 0.05 was considered statistically significant. Statistical analyses were performed using R statistics (v 3.5.1).

## RESULTS

Seventy‐four patients (55% male) were included with an median age of 63 years old (IQR 57–73). In four patients, 24 h timepoint was missed because indwelling arterial catheter was removed. Patients had a SOFA score of 8.5 (IQR 7–11). Most patients came from surgical departments (62%). Forty‐one patients (55%) fulfilled the sepsis criteria. Baseline characteristics are given in Table 2. Median haemoglobin level at inclusion was low (6.8 g/dl) and increased after transfusion (7.9 g/dl, *p* < 0.001). Septic patients were more often female and had a longer ICU admission duration at time of inclusion compared to non‐septic patients.

**TABLE 2:** Baseline characteristics of 74 critically ill non‐bleeding patients receiving a RBC transfusion

Characteristic	Total (*N* = 74)	No sepsis (*N* = 33)	Sepsis (*N* = 41)	*p*‐value
Sex, male (#, %)	41 (55)	23 (69.7)	18 (44.0)	0.03
Age, years (median, IQR)	63 (57–73)	63 (57–72)	64 (57–75)	0.56
Surgical (#, %)	46 (62)	20 (61)	26 (63)	0.99
SOFA score at inclusion (median, IQR)	8.5 (7–11)	9 (7–10)	8 (7–11)	0.76
Haemoglobin level at inclusion (g/dl) (median, IQR)	6.8 (6.3–7.4)	6.8 (6.5–7.4)	6.6 (6.3–7.4)	0.80
Days at ICU at inclusion (median, IQR)	11 (4–17)	4 (2.8–15.0)	13.5 (7.3–19.5)	0.00
Hospital mortality (#, %)	18 (24.7)	5 (15.2)	14 (34.1)	0.11
Age of transfusion unit, days (median, IQR)	13 (7–22)	13 (6–22)	16 (8–21)	0.43

*Note*: Subgroups of septic and non‐septic patients based on Sepsis‐3 criteria.

Abbreviations: ICU, intensive care unit; SOFA, sequential organ assessment score.

### RBC transfusion resulted in an increase in vWF antigen, but not in other markers

VWF antigen levels were high at baseline (median 478% [IQR 338–597]) and increased significantly 24 h after RBC transfusion (median 526% [IQR 395–623]; *p* = 0.009). VWF levels did not further increase 48 h after transfusion. Its cleaving protease ADAMTS13 was very low at baseline but was not affected by the transfusion. VWF/ADAMTS13 ratio was not affected by the transfusion. Levels of soluble ICAM‐1 and soluble TM were high at baseline but did not change significantly after transfusion. Levels of soluble syndecan‐1 were low and did not increase significantly after transfusion. Concentration of IL‐6 and TNF‐alpha did also not significantly change (Table 3).

**TABLE 3:** Level of biomarkers of endothelial cell activation and inflammation before transfusion, within hour after transfusion and 24 h after transfusion

Biomarker	Reference value	Before transfusion median (IQR)	1 h after transfusion median (IQR)	24 h after transfusion median (IQR)	*p*‐value
vWF ag (%)	50%–150%	478 (338–597)	481 (348–614)	526 (395–623)	0.009
ADAMTS13 ag (%)	50%–150%	40.4 (31.8–53.6)	43.9 (31.7–54.7)	40.8 (31.4–52.2)	0.06
vWF/ADAMTS13 ratio		11.6 (7.2–18.0)	11.7 (7.0–18.1)	12.1 (8.4–17.2)	0.98
sICAM‐1 (ng/ml)	60–218	462 (324–605)	448 (334–601)	453 (337–609)	0.83
sTM (ng/ml)	0.5–5.7	7.6 (5.9–11.3)	7.6 (5.4–11.2)	7.5 (5.2–11.1)	0.26
Syndecan‐1 (ng/ml)	50–100	2.9 (2.3–3.6)	2.9 (2.3–3.7)	2.9 (2.1–3.7)	0.94
TNFa (pg/ml)	0–16	11.5 (9–15.3)	12 (8.7–16)	12.5 (9–14.8)	0.09
IL‐6 (pg/ml)	5–15	46.2 (18.2–75.3)	43.3 (18.1–71.4)	35.2 (18.2–64.4)	0.32

*Note: p*‐value based on Friedman test.

Abbreviations: ADAMTS‐13, a disintegrin and metalloproteinase with a thrombospondin type 1 motif, member 13; IL‐6, interleukine‐6; sICAM‐1, soluble intercellular adhesion molecule 1; sTM, soluble thrombomodulin; TNF, tumour necrosis factor; vWF, von Willebrand factor.

D‐dimer and fibrinogen levels were elevated at baseline, but did not increase after transfusion. Platelet count, PT and APTT were in between reference values and also did not significantly change after transfusion (Table 4).

**TABLE 4:** Level of coagulation markers before and 24 h after transfusion

Biomarker	Reference value	Before transfusion median (IQR)	24 h after transfusion median (IQR)	*p*‐value
Platelets (10̂9/L)	150–400	186 (114–269)	199 (110–289)	0.9
D‐dimer (mg/L)	0–0.5	5.54 (3.27–9.35)	4.63 (2.82–9.35)	0.45
Fibrinogen (mg/ml)	1.5–4.0	5 (4.35–6.65)	5.2 (4.6–6.9)	0.36
APTT (s)	30–40	34 (26–46.5)	30 (25–43)	0.08
PT (s)	9.5–13.5	11.6 (11.1–12.6)	11.6 (10.9–12.3)	0.35

*Note: p*‐value based on Wilcoxon signed rank test.

Abbreviations: APTT, activated partial thromboplastin time; PT, prothrombin time.

### The inflammatory state of the recipient did not impact the effect of a RBC transfusion

**FIGURE 1:**
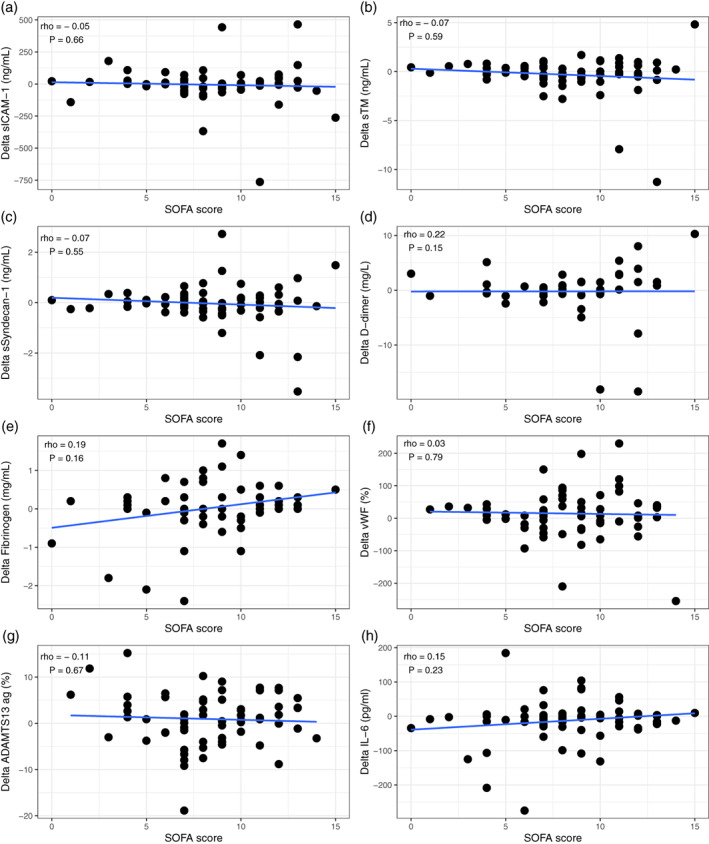
(a–h) correlation plots between post transfusion change in biomarker level and sequential organ failure assessment (SOFA) score. Rho = spearman rank correlation. TM = thrombomodulin. vWF = von Willebrand factor

Septic patients had higher baseline levels of soluble Syndecan‐1, vWF antigen and d‐dimer compared to non‐septic patients, whereas ADAMTS13 antigen levels were lower (*p* < 0.05). No differences were seen between the two groups in post transfusion change of markers of endothelial cell activation or coagulation (data not shown). In addition, post transfusion change of markers did not correlate with organ injury as assessed with the SOFA score (Figure 1).

## DISCUSSION

This study investigated the effect of a RBC transfusion on several markers of inflammation, endothelial cell activation and coagulation in the critically ill patient. Before transfusion, levels of vWF antigen, sICAM‐1 and sTM were already high, indicating that the vascular endothelium is activated in the critically ill patient [22]. D‐dimer and fibrinogen levels were also elevated at baseline. We found that RBC transfusion is associated with an increase in circulating vWF antigen levels, independent of the presence of sepsis or organ injury level of the patient. The other biomarkers did not show a post transfusion change.

So far, only one study has studied the effect of RBC transfusion on vWF antigen levels. In cardiology patients, an increase in vWF antigen levels was not observed directly following RBC transfusion [23]. However, as vWF release takes some time following a stimulus this time point may have been too short after transfusion [24]. We think it is unlikely the increase of vWF is caused by vWF that was present in RBC units, since increase was not observed immediately after transfusion but after 24 h. Most likely, RBC transfusion led to shedding of vWF from endothelial cells.

vWF antigen plays an important role in arterial and venous thrombus formation [15, 25, 26]. The increase in circulating vWF antigen levels after RBC transfusion can therefore potentially explain the increase in thrombo‐embolic events found after transfusion [4, 27]. However, we did not find an effect on markers of disseminated intravascular coagulation (DIC), such as a decreased platelet count, which is in line with earlier research [28]. Furthermore, the vWF/ADAMTS13 ratio did not increase. Therefore, the impact of 1 RBC unit on the development of thrombosis seems minimal. However, patients often receive multiple transfusions over time during their ICU stay. Whether multiple transfusions result in a more pronounced increase of vWF, followed by changes in DIC markers or the vWF/ADAMTS13 ratio should be investigated in a future study. The occurrence of thrombosis in critically ill patients is associated with worse outcomes [29], therefore more knowledge on the mechanism behind the association between RBC transfusion and thrombosis can be of great importance. By unravelling the mechanism responsible for transfusion related adverse events we may identify the responsible compounds in the RBC unit [30], enabling improvement of the transfusion product or protocols.

We did not observe an increase in biomarkers of endothelial cell damage such as syndecan‐1 (a marker of glycocalyx degradation) and thrombomodulin (a glycoprotein on the endothelial cell membrane released into the circulation by endothelial damage), suggesting that RBC transfusion leads to activation but not to endothelial cell damage. However, soluble ICAM‐1, which is also a marker of endothelial cell activation, did not increase after transfusion. A possible explanation is the high baseline level of soluble ICAM‐1 before transfusion in the critically ill patient. The expression of ICAM‐1 on the cell surface may already be saturated and therefore a further increase in the shedding of soluble ICAM‐1 after transfusion was not possible. Furthermore, in preterm neonates, an increase in sICAM‐1 was seen after the third RBC transfusion and not after the first or second [31]. Again, the impact of one RBC unit may have been too small to cause an increase in sICAM‐1 in this study.

Sepsis is a risk factor for the development of TRALI [5], however, in this study, sepsis did not impact the effect of RBC transfusion on the measured biomarkers. Possibly, biomarkers were already too much deranged due to the critical illness before transfusion.

This study has several limitations. First, this study was not powered to correlate the increase of vWF antigen levels to transfusion‐related adverse event, such as thrombo‐embolic events. Also, as ICU patients undergo multiple interventions during their stay at the ICU, it is possible that the increase in vWF occurs in all ICU patients, regardless of the transfusion of RBCs. We did not include a control group to exclude this possibility. However, as vWF levels did not further increase at 48 h, it appears unlikely that this observed increase reflects a natural trajectory in these patients.

In conclusion, RBC transfusion in critically ill patients is associated with an increase of circulating vWF antigen levels, suggesting a further increase in activation of the endothelium. This finding was independent of the presence of sepsis or organ injury level. The clinical impact of the increase of vWF cannot be distracted from this study. Our findings highlight the importance for future research to focus on the association between multiple RBC transfusions, mediators of endothelial activation such as vWF increase, and the occurrence of adverse events.

## CONFLICT OF INTEREST

The authors declare that they have no conflicts of interest.
